# Therapeutic alliance and suicidal ideation in brief cognitive behavioral therapy for outpatients

**DOI:** 10.1186/s12888-024-06205-0

**Published:** 2024-10-30

**Authors:** Laura Melzer, Thomas Forkmann, Sören Friedrich, Tobias Teismann

**Affiliations:** 1 Mental Health Research and Treatment Center, Faculty of Psychology, Ruhr-Universität Bochum, Massenbergstraße 9-13, 44787 Bochum, Germany; 2 Department of Clinical Psychology and Psychotherapy, University of Duisburg-Essen, Essen, Germany; 3 Department of Psychology, Albert-Ludwigs-Universität Freiburg, Freiburg im Breisgau, Germany

**Keywords:** Therapeutic alliance, Working alliance inventory, Suicidality, Suicidal ideation

## Abstract

**Objective:**

The therapeutic alliance is central to psychotherapy. However, research on the relationship between alliance and suicidality is scarce. We examined whether pretreatment suicidality is associated with an impaired alliance formation in brief cognitive behavioral therapy (CBT) and whether the therapeutic alliance is associated with change in suicidal ideation (difference between pretreatment and posttreatment assessment).

**Methods:**

*N* = 643 outpatients (64% female; age: *M*[*SD*] = 37.09[13.15], range: 18–73 years), received 12 sessions of manual-based short-term CBT for primary diagnosis treatment. Using self-report questionnaires, suicidal ideation and behavior were assessed before and after therapy, patient-rated therapeutic alliance was assessed after the fourth session and posttreatment. We performed correlation analyses and two hierarchical linear regressions, unadjusted and adjusted for possible confounding variables (age, gender, lifetime suicide attempts and depression).

**Results:**

Pretreatment suicidal ideation was not predictive of the quality of the early alliance. In addition, the therapeutic alliance was not predictive of change in suicidal ideation.

**Conclusions:**

In the outpatient setting, no association was found between therapeutic alliance and suicidality. Future studies should investigate therapeutic alliance as a predictor of prospective suicidal behavior in different clinical settings.

## Introduction

The prevention of suicide is considered a significant public health concern [1, 2]. Suicide prevention research focuses on strategies that help reduce the risk of suicide [3], targeting traditional risk factors such as suicidal ideation and behavior [4]. A recent meta-analysis demonstrated that psychotherapeutic interventions effectively reduced patients’ suicidal ideation rates and number of suicide attempts [5]. However, the precise mechanisms underlying effective psychotherapeutic suicide prevention are not yet fully understood [6].

One of the most studied process variables in psychotherapy is the therapeutic alliance (TA), which is a robust predictor of treatment success [7] and a mediator of therapeutic outcomes [8]. According to Bordin [9], the therapeutic alliance consists of three components: (1) patient and therapist collaboratively agree on goals, as well as (2) on interventions and (3) form an affective bond over the course of therapy. With regard to the treatment of suicidal patients, the role of the therapeutic relationship has been studied rather rarely and findings are heterogeneous [10, 11]. On the one hand, there are several studies showing that clinicians have negative views of suicidal patients [12, 13] and on the other hand, pretreatment suicidal ideation and behavior do not seem to have a negative impact on the quality of the formation of a therapeutic alliance [11]. Moreover, there are studies showing that a good therapeutic alliance has an antisuicidal effect, i.e., is predictive of an amelioration of suicidal ideation over the course of psychotherapy [e.g., 14, 15, 16, 17], whereas other studies find no relation between the therapeutic alliance and the improvement of suicidal ideation within psychotherapy [18, 19]. Accordingly, it appears that the therapeutic alliance has antisuicidal properties in the context of some forms of treatment but not in others [cf. 20]. Thus, it seems important to examine the relevance of the therapeutic alliance in the context of different types of treatments and settings. Previous studies have primarily examined the therapeutic alliance as part of suicide-specific psychotherapies and in the treatment of inpatients [10, 11]. There is a lack of research on outpatient psychotherapy in routine care, despite the high prevalence of suicidal ideation in this treatment setting [21].

Against this background, the first aim of the present study was to examine the extent to which current suicidal ideation and lifetime suicide attempts were negatively related to the formation of a therapeutic alliance. The second aim was to investigate the extent to which the early therapeutic alliance was associated with a change in suicidal ideation in a mixed sample of outpatients who received brief cognitive-behavioral therapy in routine care, that was not suicide-specific but rather tailored at the patients’ primary diagnoses. Differences in patient characteristics might moderate this association. Based on previous studies, we expected that demographics such as age and gender would have a moderating effect: Matching therapist and client characteristics is associated with better outcomes [22]. Older men may be reluctant to disclose suicidal ideation to younger female therapists [23], but a “youth effect” may also lead to better quality of the therapeutic alliance [24]. As results on moderation effects of depression [25–27] and history of suicide attempts [27, 28] from previous studies are mixed, we aimed at exploratively investigating potential effects on the relation between alliance and change in suicidal ideation. We therefore assessed age, gender, history of suicide attempts and depressive symptoms as potential confounding variables.

## Methods

### Participants & procedure

A total of *N* = 643 patients (64% female; age: *M*[*SD*] = 37.09[13.15], range: 18–73 years), undergoing cognitive-behavioral therapy (CBT) in a German outpatient clinic between March 2017 and May 2023 were included in the current study for secondary data analysis. Data collection in the outpatient clinic was interrupted during the COVID-19 pandemic. Thus, there is no data between 2020 and 2021, except for two cases. Inclusion criteria were a complete pre- and posttreatment assessment of suicidal ideation and therapeutic alliance, and an age of at least 18 years, as therapeutic alliance was only assessed in adults outpatients. There were no exclusion criteria. The initial sample comprised 1667 individual patients who completed the pretreatment assessment. Of those, 828 patients (49.6%) took part in an early treatment assessment at session four, and 643 patients (38.5%) completed a posttreatment assessment after session twelve. According to the International Classification of Diseases (ICD) [29], patients were most commonly diagnosed with neurotic, stress-related and somatoform disorders (ICD-10, F4: 58%), affective disorders (ICD-10, F3: 29%) or personality disorders (ICD-10, F6: 9%). Concurrent suicidal ideation assessed with the Suicide Ideation and Behavior Scale (SIBS) [30] was reported by *n* = 273 patients (42% with SIBS score ≥ 1) at the pretreatment assessment. Of those, 175 patients (64.1%) reported a reduction in their suicidal thoughts, 43 patients (15.8%) reported no change, and 55 patients (20.1%) reported an increase. 11% of the patients (*n* = 69) reported a history of lifetime suicide attempts and one patient attempted suicide during treatment as stated. All patients were informed about regular assessments at the clinic for both diagnostic and research purposes. All patients provided informed consent and completed questionnaires at the pretreatment assessment (T1) and again after 12 therapy sessions (posttreatment, T2). This latter assessment was conducted approximately five months (mean [*M*] = 23.77 weeks, standard deviation [*SD*] = 8.79) after the first session. Forty-six clinical psychologists (*n* = 39 female therapists [84.8%]; *n* = 7 male therapists [15.2%]) conducted manual-based therapies to treat the initial diagnosis, e.g. depression or anxiety treatment. All therapists were psychologists in advanced CBT-training and provided treatment under supervision and in accordance with treatment guidelines. The local ethics committee approved the data collection (318/2016).

### Measures

All self-report measures were administered before treatment (T1), except for the Working Alliance Inventory, which was administered at session four (early therapy), and again after session 12 (T2). The therapist-rated version was not assessed.

*Working Alliance Inventory* (WAI) [31]. The WAI measures three factors of the alliance according to Bordin’s [9] pantheoretical model: Bond, Tasks and Goals. The German patient-rated version of the revised short form (WAI-SR) [32] with 12 items was used in the present study. The three subscales consisted of four items each on a 5-point Likert scale ranging from “1 = seldom” to “5 = always”, that is the goal subscale (e.g., “My therapist and I collaborate on setting goals for my therapy”), the bond subscale (e.g., “I believe my therapist likes me”) and the task subscale (e.g., “What I am doing in therapy gives me new ways of looking at my problem”). As in previous studies [14, 32], only the total score was calculated with higher scores indicating a stronger alliance. The internal consistency of the total scale was excellent in an inpatient (*α* = 0.93) and an outpatient sample (*α* = 0.90) [33] as well as in the present study (pretreatment: *α* = 0.89; posttreatment: *α* = 0.91).

*Suicide Ideation and Behavior Scale* (SIBS) [30]. The SIBS is a German self-report instrument that assesses suicidal ideation and behavior within the past four weeks. Six items refer to the frequency of suicidal thoughts on a 6-point Likert scale ranging from “0 = never” to “5 = many times every day”. Two further items dichotomously (yes/no) assess suicide attempts within the past four weeks and lifetime suicide attempts. The SIBS has demonstrated good internal consistency (*α* = 0.92) in the validation study [30] as well as the present study (pretreatment: *α* = 0.83; posttreatment: *α* = 0.86). Change in suicidal ideation was calculated as the difference between pretreatment and posttreatment total score.

*Beck Depression Inventory* (BDI-II) [34]. The BDI measures the severity of depression using 21 items. Patients respond to a 4-point Likert scale ranging from 0 to 3 based on a two-week time period. The internal consistency was shown to be good in German clinical samples (α ≥ 0.84) [35] and excellent in the present study (pretreatment: α = 0.92; posttreatment: α = 0.93).

### Statistical analyses

Statistical analyses were conducted with SPSS 28. Descriptive statistics and zero-order bivariate correlations between study variables were calculated. Changes in study variables between pretreatment and posttreatment scores were analyzed using t-tests for dependent samples and effect sizes *d* [36]. To evaluate the association between pretreatment suicidal ideation and the early alliance, a 3-step hierarchical linear regression analysis was conducted with working alliance as the dependent variable. Demographics (age, gender) and depression were included as potential confounders in a first regression model. A second model additionally controlled for lifetime suicidal attempts. Suicidal ideation was included as the predictor of interest in a third model. To evaluate the association between early alliance and post-treatment suicidal ideation, another 3-step hierarchical linear regression analysis was conducted with change in suicidal ideation (difference between pretreatment and posttreatment) as the dependent variable and age, gender, and pretreatment depression were included as potential confounding variables in a first model. A second model controlled for lifetime suicidal attempts and in a third model, early working alliance was included as predictor variable. For our primary analyses, power analyses using the G*Power program, version 3.1 [37] indicated that the sample size was sufficient to detect even small effects of *f*^2^ ≥ 0.02 (post hoc sensitivity calculation for *n* = 643, power = 0.80, *α* = 0.05) and for valid results (power > 0.80, *α* = 0.05, effect size: *f*^*2*^ = 0.15) [cf. 38].

## Results

Table 1 shows descriptive and change statistics for all study variables. Findings point to significant changes in all variables from pre- to posttreatment with small to medium effect sizes regarding the reduction of suicidal ideation and depression and moderate increases in therapeutic alliance ratings.

**Table 1:** Descriptive and change statistics (*N* = 643)

Variable	Pretreatment M(SD)	Posttreatment M(SD)	t	*p*	d
WAI	47.7 (7.4)	49.9 (7.0)	*t*(642) = -9.6	0.000	0.37
SIBS	1.6 (3.2)	1.2 (2.9)	*t*(642) = 4.0	0.000	−0.16
BDI-II	22.2 (12.2)	17.2 (11.6)	*t*(642) = 13.6	0.000	−0.52

Note. *M* = mean; *SD* = standard deviation; WAI = Working Alliance Inventory, Short-Form Revised, Patient-rated; SIBS = Suicide Ideation and Behavior Scale; BDI-II = Beck Depression Inventory

Table 2 shows correlations between all study variables. There were significant negative associations between pretreatment suicidal ideation and the therapeutic alliance at session four and at the posttreatment assessment. Lifetime suicide attempts did not significantly correlate with the patient-rated therapeutic alliance.

**Table 2:** Intercorrelation between study variables (*N* = 643)

Measure	1.	2.	3.	4.	5.	6.	7.	8.	9.
1. WAI_pre_	-								
2. WAI_post_	0.73^**^	-							
3. SIBS_pre_	− 0.14^**^	− 0.13^**^	-						
4. SIBS_post_	− 0.12^**^	− 0.16^**^	0.63^**^	-					
5. BDI-II_pre_	− 0.10^**^	− 0.13^**^	0.51^**^	0.40^**^	-				
6. BDI-II_post_	− 0.09^*^	− 0.20^**^	0.38^**^	0.53^**^	0.67^**^	-			
7. Age	− 0.05	− 0.08^*^	− 0.14^**^	− 0.12^**^	− 0.02	0.52	-		
8. Sex	0.10^*^	0.09^*^	− 0.05	− 0.03	0.09^*^	0.08^*^	− 0.02	-	
9. SA_life_	− 0.03	− 0.04	0.24^**^	0.22^**^	0.18^**^	0.21^**^	.-0.01	0.06	
10. SI_change_	− 0.02	− 0.04	0.50^**^	− 0.19^**^	0.19^**^	− 0.07	0.01	− 0.05	0.13^**^

Note. *M* = mean; *SD* = standard deviation; WAI-SR = Working Alliance Inventory, Short-Form Revised, Patient-rated; SIBS = Suicide Ideation and Behavior Scale; BDI-II = Beck Depression Inventory; pre = pre-therapy; post = post-therapy; SAlife = lifetime Suicide Attempts; SIchange = Change in Suicidal Ideation (difference between pretreatment and posttreatment)

**Significant at *α* = 0.01. *Significant at *α* = 0.05

Pretreatment suicidal ideation did not significantly predict the quality of the early therapeutic alliance, controlling for lifetime suicide attempts, age, gender and pretreatment depression. Female gender and lower levels of pretreatment depression were associated with a better therapeutic alliance at session four, explaining 3% of variance in Model 1 (see Table 3).

**Table 3:** Hierarchical regression analysis for variables predicting therapeutic alliance at session four (*N* = 643)

	First Model			Adjusted for history of suicide attempts			Full model		
Variable	*B*	*SE B*	*p*	*B*	*SE B*	*p*	*B*	*SE B*	*p*
Intercept	49.50	1.08	**< 0.001**	49.50	1.08	**< 0.001**	49.49	1.08	**< 0.001**
Age	− 0.03	0.02	0.117	− 0.03	0.02	0.117	− 0.03	0.02	0.108
Gender	1.68	0.61	**0.006**	1.68	0.61	**0.006**	. 1.65	0.61	**0.007**
BDI-II_pre_	− 0.07	0.02	**0.004**	− 0.07	0.02	**0.004**	− 0.06	0.03	**0.020**
SA_life_				0.44	0.02	0.648	0.58	0.98	0.557
SI_pre_							− 0.08	0.10	0.416
*R*^2^/ *R*^2^ adjusted	0.026/0.021			0.026/0.020			0.027/0.020		
*F* for change in *R*^2^	5.690**			4.314*			3.582*		

Note. BDI-II = Beck Depression Inventory. Pre = pretreatment. SA_life_ = History of suicide attempts. SI = Suicidal ideation

*p < .05, **p < .001

Furthermore, the quality of the therapeutic alliance at session four was not predictive of changes in suicidal ideation, while controlling for lifetime suicide attempts, depression, age and gender. Only depression and history of suicide attempts were predictive of changes in suicidal ideation (see Table 4), marginally increasing the explained variance from 2% in the first model to 4% in the second model that included confounding variables.

**Table 4:** Hierarchical regression analysis for variables predicting change in suicidal ideation (*N* = 643)

	First Model			Adjusted for history of suicide attempts			Full model		
Variable	*B*	*SE B*	*p*	*B*	*SE B*	*p*	*B*	*SE B*	*p*
Intercept	− 0.28	0.35	0.420	− 0.29	0.35	0.404	− 0.41	0.72	0.571
Age	0.01	0.01	0.523	0.01	0.01	0.409	0.01	0.01	0.404
Gender	− 0.24	0.20	0.222	− 0.25	0.19	0.201	− 0.25	0.20	0.197
BDI-II_pre_	0.03	0.01	**< 0.001**	0.02	0.01	**0.005**	0.02	0.01	**0.005**
SA_life_				1.02	0.31	**0.001**	1.02	0.31	**0.001**
WAI_pre_							0.01	0.01	0.852
*R*^2^/ *R*^2^ adjusted	0.023/0.019			0.039/0.033			0.039/0.032		
*F* for change in *R*^2^	5.047*			6.494**			5.194**		

Note. BDI-II = Beck Depression Inventory. Pre = pretreatment. SA_life_ = History of suicide attempts. SI = Suicidal ideation. WAI = Working

Alliance Inventory

*p < .05, **p < .001

All analyses were re-run including only those patients (*n* = 273) who suffered from suicidal ideation at the pretreatment assessment. There were no differences in the overall result pattern.

## Discussion

The aim of the current study was to analyze the relationship between patient-rated therapeutic alliance and suicidal ideation in German outpatients receiving brief CBT. There were two main findings: Pretreatment suicidal ideation as well as lifetime suicide attempts were not predictive of the patient-rated therapeutic alliance at session four, nor did the therapeutic alliance predict changes in suicidal ideation from pre- to posttreatment of brief cognitive behavioral therapy (CBT).

Consistent with previous studies [11], neither pretreatment suicidal ideation nor lifetime suicide attempts interfered with the development of the therapeutic alliance. As such, patients and therapists were able to form a therapeutic alliance early on in outpatient psychotherapy, regardless of the occurrence of suicidal ideation and/or a history of suicide attempts. This is a positive finding. However, it should be noted that very few patients in the current study suffered from extreme suicidal ideation and/or had to be treated immediately after a suicide attempt [cf. 13]. The study was conducted on data from a mixed sample of outpatients receiving CBT tailored at their primary diagnoses. Thus, treatment was in most cases not specifically addressing suicidality. Different from some [14–17], but not all [18, 19] previous studies, the therapeutic alliance was not predictive of changes in suicidal ideation from pre- to posttreatment in brief CBT. With regard to the reduction of suicidal thoughts, outpatients in routine care CBT thus appear to benefit regardless of the quality of the therapeutic alliance. Our result underlines the need to investigate the role of the therapeutic alliance for suicidal thoughts in different treatment settings [cf. 10, 11]. One might speculate that in routine care outpatient psychotherapy – and when working with less suicidal patients – therapeutic interventions play a greater role regarding the amelioration of suicidal ideation than process variables such as the therapeutic alliance [cf. 39]. At the same time, it should also be noted that some of the previous studies that found significant relations between suicidal ideation and therapeutic alliance examined longer follow-up periods [e.g., 15, 27, 35, 40] than the present study. This should be taken into account in future studies.

Several limitations must be considered when interpreting the results of the present study. First, only the patients’, but not the therapists’ perspective on the therapeutic alliance was assessed in the current study. Although previous research suggests that the therapist’s perspective on the therapeutic relationship is less related to treatment success than the patient’s perspective [7, 16, 41], future studies should nevertheless consider both perspectives simultaneously. It is possible that an overlap between the two perspectives may be relevant. Second, generalizability to other psychotherapy routine care facilities is limited given the fact that all treatments in the current study had a cognitive-behavioral focus and were delivered in a university outpatient clinic. Third, moderating effects of other treatments, such as medication use could not be controlled for. Medication is recorded by clinicians, but not collected for research data. Fourth, the information about suicidal ideation was self-reported and, thus, subject to potential recall bias and denial. Fifth, only posttreatment suicidal ideation data were available in the present study, while no follow-up data were available to assess the long-term effect of the therapeutic alliance on reducing suicidal thoughts [cf. 15, 33]. Sixth, it should be noted that the therapists were rather young and inexperienced. It is unclear to what extent therapists in the present study paid particular attention to establishing a good therapeutic relationship with suicidal patients [cf. 42]. Following suggestions from prior studies, future research should examine the influence of therapist characteristics on the formation and maintenance of the therapeutic alliance in the context of discussion suicidal thoughts and acts in psychotherapy [11].

## Conclusion

In a routine care outpatient setting, no association was found between the therapeutic alliance and suicidal ideation. This suggests that suicidal patients are not reluctant but are just as successful as non-suicidal patients in establishing a therapeutic alliance; and that therapists are just as successful in establishing a good therapeutic relationship with suicidal patients. Furthermore, in the context of routine care psychotherapy, the reduction of suicidal thoughts does not seem to depend on a close therapeutic relationship. However, this should not be misinterpreted to mean that a therapeutic relationship is not important for suicidal patients in general [43].

## Data Availability

All relevant data are reported within the paper. The datasets are available from the corresponding author on reasonable request.

## Abbreviations

CBT: Cognitive Behavioral Therapy
ICD: International Classification of Diseases
SA: Suicide Attempts
SI: Suicidal Ideation
SIBS: Suicide Ideation and Behavior Scale
WAI: Working Alliance Inventory
WAI: SR–Working Alliance Inventory–Short–form revised
BDI: II–Beck Depression Inventory
